# The upper limit and lift force within inertial focusing in high aspect ratio curved microfluidics

**DOI:** 10.1038/s41598-021-85910-2

**Published:** 2021-03-19

**Authors:** Javier Cruz, Klas Hjort

**Affiliations:** Division of Microsystems Technology, Uppsala University Ångström Laboratory, Uppsala, Sweden

**Keywords:** Biomedical engineering, Applied microbiology, Nanobiotechnology, Microfluidics

## Abstract

Microfluidics exploiting the phenomenon of inertial focusing have attracted much attention in the last decade as they provide the means to facilitate the detection and analysis of rare particles of interest in complex fluids such as blood and natural water. Although many interesting applications have been demonstrated, the systems remain difficult to engineer. A recently presented line of the technology, inertial focusing in High Aspect Ratio Curved microfluidics, has the potential to change this and make the benefits of inertial focusing more accessible to the community. In this paper, with experimental evidence and fluid simulations, we provide the two necessary equations to design the systems and successfully focus the targets in a single, stable, and high-quality position. The experiments also revealed an interesting scaling law of the lift force, which we believe provides a valuable insight into the phenomenon of inertial focusing.

## Introduction

Inertial focusing is a phenomenon that enables focusing of initially randomly distributed particles in a fluid into well-defined positions within microfluidic channels, thereby facilitating the detection, isolation and analysis of rare targets of interest in complex fluid samples like blood, for instance. The technology has attracted much attention over the last decade thanks to its attributes; it allows for high through-put focusing, concentration and separation of particles with high resolution, it does not require labelling of the targets, it works for neutrally buoyant particles, and the operation of the systems is relatively simple (the sample simply has to pass through the microchannel at a controlled flow rate)^1–3^. With such a promising performance, the technology has grown rapidly.

The phenomenon has been physically and analytically described^4–9^ since it was first observed by Segré and Silberberg in 1961^10^. Migration and focusing of particles occur in microfluidic systems where inertia is not negligible, and it is attributed to a net lift force (${F}_{L}$). The net lift force is composed of a shear-gradient induced lift force that pushes particles from the center of the channels towards the walls, and a wall-induced lift force, which repels particles away from the walls^1,2,4^. The net lift force is often complemented by the drag of a secondary flow (${F}_{D}$) to reduce the number of focus positions and tune their location^1,2^. Systems with different configurations and cross sections have been explored aiming at tailoring the force fields and achieve different performances. With it, multiple successful applications of the phenomenon have been presented, such as isolation and extraction of circulating tumour cells (CTC) from blood samples^11,12^ and focusing, separation and concentration of pathogenic bacteria from water samples^13,14^. Modern reviews covering the physics, the performances from different configurations and cross sections, and successful applications can be found in the literature^1–3,15–18^.

**Figure 1:**
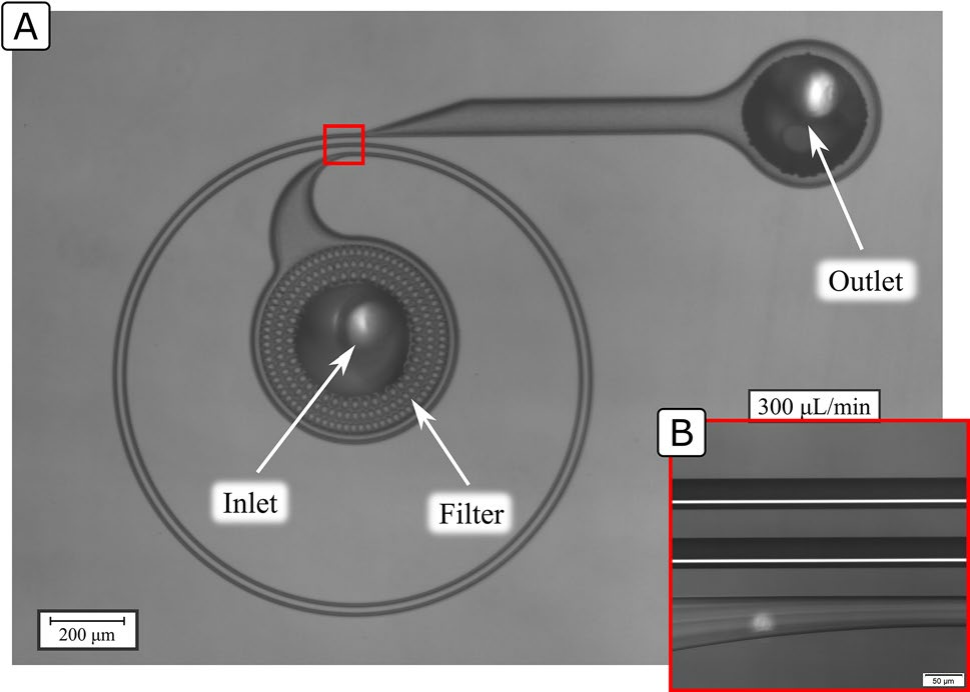
(**A**) Example of HARC system under the microscope. The red mark indicates the location where the high magnification pictures were taken for the evaluation of the performance of different devices. (**B**) Example of performance with 4 µm polystyrene particles from a HARC system with two loops, 41 × 84 µm ($WxH$) and $R$ 25 mm.

A notable limitation for the technology is the rapid increase in the pressure needed to run the systems when targeting submicron particles^13^. Although robust silicon-glass systems have been shown to withstand up to 200 bar and allow for focusing particles down to 0.5 µm^14^, smaller particles of interest such as viruses or exosomes remain out of reach. Another major limitation is the fact that the position of the focused particles (focus position) generally depends on multiple variables. In fact, the focus position generally shifts in tortuous ways as a function of all variables defining the system and the flow: the width ($W$), depth ($H$), radius ($R$) and shape of the microchannel, the maximum speed of the flow (${U}_{m}$) and the particle hydrodynamic size ($a$; defined as the diameter in case of spherical particles)^3,19^. While this is the source of the potential for particle separation, it also makes designing the systems difficult and limits their practical application as extremely fine tolerances are needed both in the fabrication of the systems and control of the flow rate ($Q$) during the operation. Because of this, engineering systems that exploit inertial focusing for practical applications remains challenging for those in the field and inaccessible for those outside. With this paper, extending our initial work^20^, we aim at contributing to the field by making inertial focusing more accessible and hopefully allow more in the scientific community to benefit from its outstanding performances. We recently presented inertial focusing in High Aspect Ratio Curved (HARC) microchannels^20^, a line of inertial focusing that overcomes the major limitation of the shifting focus positions. The systems consist of curved rectangular microchannels with an aspect ratio $AR=H/W>1$, in which the force field induced by the combination of ${F}_{L}$ and ${F}_{D}$ leads to all particles focusing into a single position that is stable within a wide range of flow rate. An example of HARC system built in silicon-glass is shown in Fig. 1A, and an example of focus performance in Fig. 1B.

**Figure 2:**
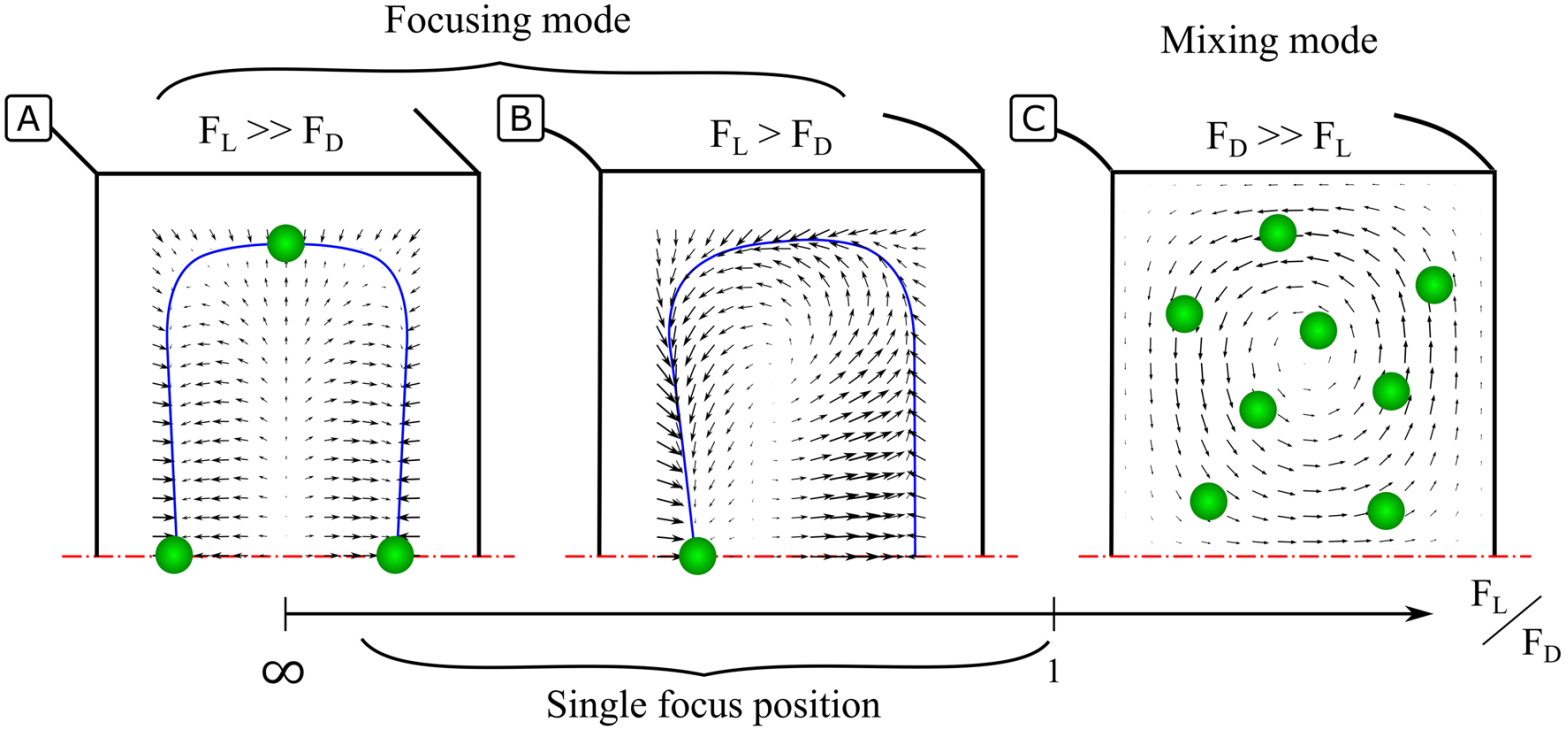
Generic force fields resulting from the combination of ${F}_{L}$ and ${F}_{D}$ in inertial focusing systems with high aspect ratio. The equilibrium perimeter (EP) is represented with a blue line. (**A**) Straight system. Only ${F}_{L}$ is present, leading to four focus positions. (**B**) Curved system where both forces are present and ${F}_{L}>{F}_{D}$. Particles focus into a single position. (**C**) Curved system where both forces are present and ${F}_{D}>>{F}_{L}$. Particles follow the vortexes indefinitely and remain unfocused.

Figure 2 shows different force fields in HARC microchannels depending on the ratio of the two forces. The extreme case where the force field consists purely of ${F}_{L}$ is similar to a straight microchannel; a focus position is achieved at the centre of each wall. Figure 2A illustrates an example of such a force field as calculated by Liu^21^. The other extreme, consisting purely of ${F}_{D}$, leads to particles following two symmetrical vortexes indefinitely, remaining randomly distributed. An example of such a force field is shown in Fig. 2C as calculated by COMSOL Multiphysics. In an intermediate regime, however, where both forces are relevant but ${F}_{L}>{F}_{D}$ at the central region close to the inner wall, the force field resulting from their superposition brings particles to a single equilibrium position, Fig. 2B.

To succeed in engineering a HARC system and achieve a performance like that shown in Fig. 2B, two conditions must be met. First, all particles in the system should have time to reach the focus position. Stemming from this condition, there is a lower limit of $Q$ (${Q}_{min}$) for the operation of the systems, for which an equation was proposed^20^. Second, ${F}_{L}$ must be strong enough to stop the particles from crossing to the outer wall while following the vortexes of the secondary flow. Stemming from this condition, HARC systems have an upper limit of $Q$ (${Q}_{max}$) over which ${F}_{D}$ surpasses ${F}_{L}$ and particles are not focused. The mathematical expression to fulfil this condition is simply ${F}_{L}>{F}_{D}$ at the region of the inner wall. However, although much progress has been done in the field^1,2,16,17^, there is no consensus for an accurate expression for ${F}_{L}$ and, therefore, this upper limit—the last piece to enable the complete design of HARC systems—remains unknown and resolving it is the focus of this work.

In this paper, we studied the upper limit experimentally. We gathered experimental data of ${Q}_{max}$ under different conditions from devices fabricated on silicon-glass and, together with a study about the strength of the secondary flow by COMSOL Multiphysics, we propose an equation that predicts the aforementioned upper limit. With this contribution, designing inertial focusing systems that focus particles becomes easily accessible. The focus position is stable and succeeding in the focusing is reduced to fulfilling two—fairly simple—known equations; the equations for the lower and upper limit.

Last, since the experimental measurements contain information about the strength of ${F}_{L}$, the data was used to derive an equation expressing its magnitude and scaling, which we believe may provide a valuable insight into the phenomenon of inertial focusing. With a better understanding about the ${F}_{L}$, we expect the technology to reach smaller particles in the near future.

## Theory of HARC systems

In HARC systems, there is a net lift force (${F}_{L}$) similar to that in straight channels, which makes particles migrate first to an equilibrium perimeter (EP) and slowly to the centre of the faces^22,23^; Fig. 2A shows an example of such a force field (as presented by Liu^21^). Although there is no agreement on an expression for ${F}_{L}$, it is known to depend strongly on the particle size, channel geometry and flow conditions. The distribution shown in Fig. 2A can be taken as a generic shape of the force field illustrating the phenomenon.

The curvature of the system induces a secondary flow (perpendicular to the main flow) that takes the shape of two vortexes and drags particles in the direction of its streamlines^24^; Fig. 2C. The novelty in HARC systems is that these secondary streamlines are mostly tangential to the EP and particles are easily swept over it until the central part of the inner wall^20^. In that region, the secondary flow turns and finds the opposition of ${F}_{L}$, whose horizontal component acts as a barrier (Lift Barrier; ${B}_{L}$). Provided that ${B}_{L}$ is stronger than the drag by the secondary flow (${F}_{D}$), particles are stopped and focused into a single position; Fig. 2B. If, on the contrary, ${B}_{L}$ is not strong enough, particles go through and keep circulating indefinitely; Fig. 2C.

The study of inertial focusing in HARC microchannels can be divided in two sections: The collection of particles around the EP by the secondary flow, which was explained in detail in our previous work^20^, and the retention/focus of particles at the inner wall; the focus of this paper.

### Collection of particles around the cross section by the secondary flow

For particles to reach the single focus position, one Dean Loop must be completed (i.e. a full rotation of the secondary flow), which sets the lower limit of the systems. An approximation to the necessary channel length for this to happen, expressed as the number of loops (${N}_{L}$), was previously derived^20^ (Eq. 1) by considering that particles quickly fall to the EP and slide freely around it following the secondary flow while they move forward with the main flow:

1 $${N}_{L}\approx \frac{20{(AR)}^{2}}{Re}$$

where $Re$ is the Reynolds number of the channel, defined as $Re=\frac{\rho {U}_{m}W}{\mu }$, with $\rho$ and $\mu$ being the density and dynamic viscosity of the fluid, respectively.

Rearranging Eq. (1), the minimum flow rate (${Q}_{min}$) that will achieve focus for a given system with water-based samples is obtained (Eq. 2):

2 $${Q}_{min}\approx \frac{0.6{(AR)}^{3}W}{ {N}_{L}}\frac{\mu L/min}{\mu m}$$

### Retention/focus of particles at the inner wall

When particles reach the inner wall by following the secondary flow, the horizontal component of ${F}_{L}$ acts as a barrier that hinders them from crossing to the outer wall (Lift Barrier; ${B}_{L}$). Provided that it is strong enough, the horizontal component of ${F}_{D} ({F}_{Dx}$) is cancelled and the vertical one brings all particles into a single focus position at the central part. If, on the contrary, ${F}_{Dx}$ is stronger than ${B}_{L}$, particles continue following the secondary flow and remain unfocused.

Of particular interest is the fact that, for a given HARC system, increasing $Q$ makes particles eventually surpass ${B}_{L}$, defining an upper limit of flow rate (${Q}_{max}$) in the operation. This is not surprising, as ${F}_{D}$ is known to grow with ${U}^{2}$, while ${F}_{L}$ has been reported in multiple instances to grow less strongly than that^5,14,20,21,25,26^. At this particular event, ${F}_{Dx}$ transitions from being weaker to being stronger than ${B}_{L}$. In other words, at that moment, both forces can be assumed to be equal (${{F}_{Dx}=B}_{L}$) and, therefore, understanding ${F}_{D}$ at these events leads to understanding ${B}_{L}$.

**Figure 3:**
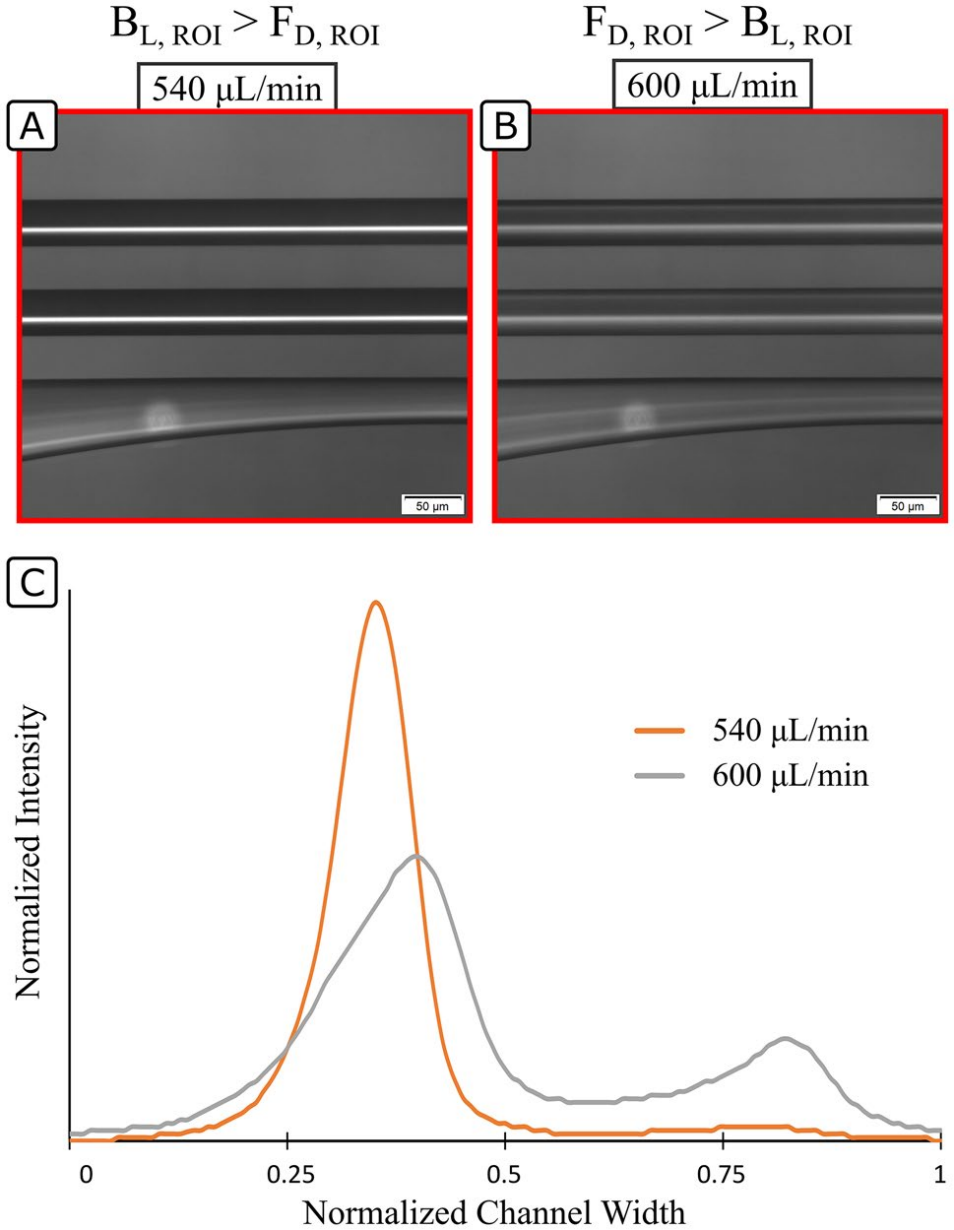
Performance of a HARC system with two loops, 41 × 84 µm ($WxH$) and $R$ 25 mm with 4 µm polystyrene particles. At 540 µL/min, the particles are stopped by the Lift Barrier and a high-quality focus line is obtained by the inner wall. At 600 µL/min, the particles are not stopped by the Lift Barrier and remain unfocused. (**A**,**B**) View under the microscope. (**C**) Intensity profile.

Figure 3 illustrates the performance of a HARC system before and after ${Q}_{max}$; Fig. 3A,B show the view under the microscope and Fig. 3C the intensity profile. It can be seen how a good quality focus is achieved at 540 µL/min ($Q<{Q}_{\mathrm{max}}$), while at 600 µL/min ($Q>{Q}_{\mathrm{max}}$) the system does not have the capacity to focus the particles any longer.

**Figure 4:**
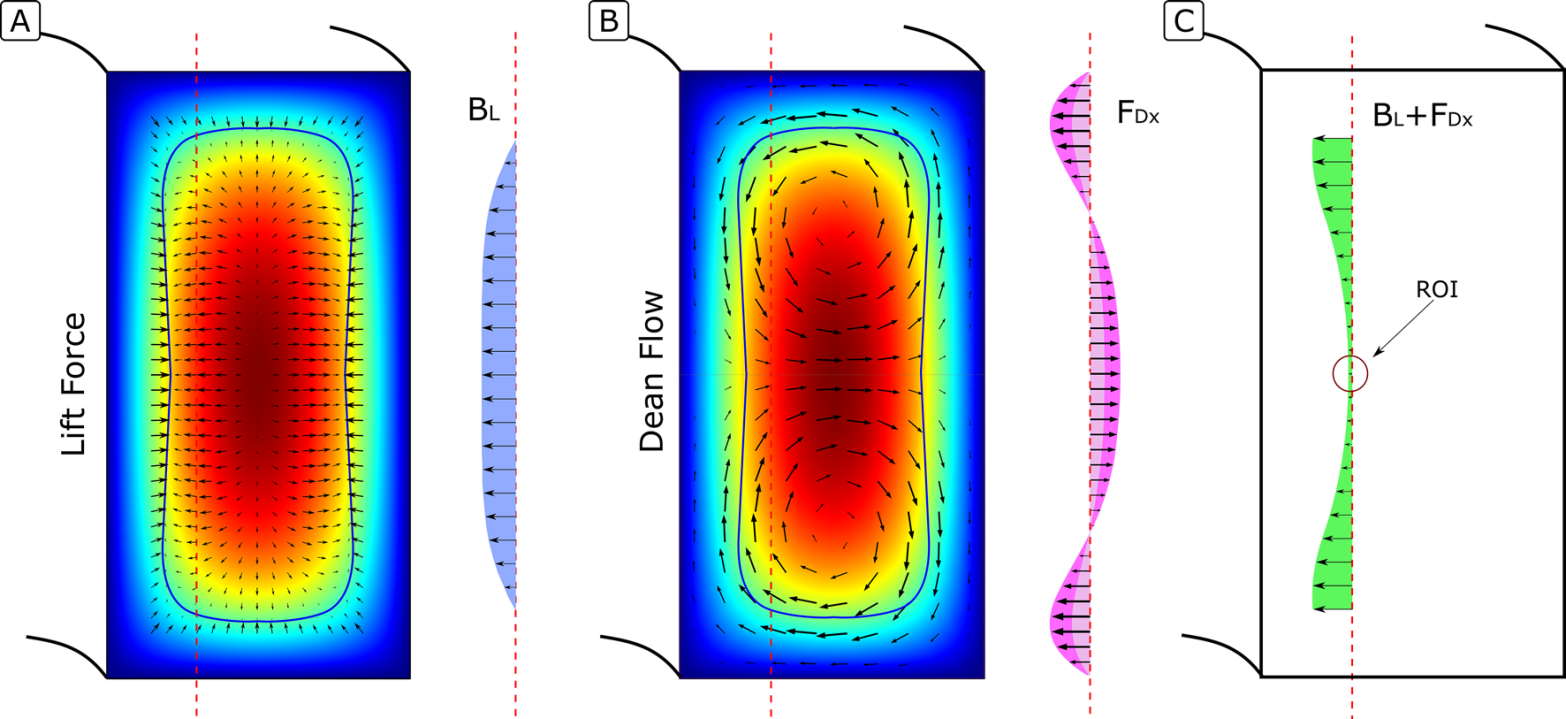
(**A**) Generic force field of ${F}_{L}$ and isolation of the horizontal component at $W/3$. (**B**) Generic force field of ${F}_{D}$ and isolation of the horizontal component at $W/3$. The different tonality of the isolated component indicates how it grows as the secondary flow gains strength. (**C**) Combination of both forces at $W/3$.

Rather than studying the force balance in the whole ${B}_{L}$, the analysis can be localized at the position where particles first breach the barrier. We identified such position to be at a distance approximately $W/3$ from the inner wall, the last position where particles focused experimentally prior to defocusing with further increase of $Q$, see Fig. 3. In the model used in this paper, further analysis of the phenomenon was done at this particular location. Figure 4A sketches the distribution of ${F}_{L}$ proposed by Liu^21^, isolating its horizontal value at $W/3$ from the inner wall (${B}_{L}$). Figure 4B sketches the distribution of ${F}_{D}$ in HARC channels obtained by COMSOL simulations, isolating its horizontal value at $W/3$ from the inner wall $({F}_{Dx}$). Last, Fig. 4C shows the result of the combination of the two, where it can be seen how a further relative increase in ${F}_{D}$ will induce a breach in ${B}_{L}$ right at the symmetry line.

The critical position, to which we refer as region of interest (ROI), is then defined at the symmetry line and $W/3$ from the inner wall, and the condition for HARC systems to focus particles (Eq. 3):

3 $${B}_{L, ROI}>{F}_{D, ROI}$$

With this, the study of the balance between the two forces in HARC systems is reduced to a single point (ROI). Expressions for both ${B}_{L, ROI}$ and ${F}_{D, ROI}$ are derived in this paper to fully describe Eq. (3) and provide an expression for the upper limit.

## Material and methods

### Device fabrication

The devices were fabricated on hybrid silicon-glass systems so they could stand up high pressures without undergoing deformation. First, the microchannels were patterned on a silicon wafer with photoresist 1813 (chromium mask; Micro Lithography Services Limited). The microchannels were dry etched with the photoresist as mask using a short cycle Bosch process to minimize the roughness of the sidewall (Tegal dry etcher; ~ 200 nm escalloping). The wafer was then cleaned and 0.5 µm of Al were sputtered to cover the channels and act as an etch stop for the next step. Lithography was done on the back side (plastic mask; Micro Lithography Services Limited) and via holes were dry etched for the fluidic connections (Tegal dry etcher; ~ 2 µm escalloping). With all the micromachining finished, the silicon wafer, together with a borosilicate wafer, was cleaned and activated in piranha solution for 15 min. Both wafers were put together and anodically bonded (380 ºC and 1 kV for 4 h). Last, glass capillaries (Genetec, 100 and 170 µm inner and outer diameter, respectively) that served as fluid connections were glued with epoxy (EPO-TEK 302-3M).

### Setup

Fluorescent polystyrene particles (0.70, 0.79, 0.92 and 1.0 µm, Thermo Scientific Fluoro-Max) were suspended in deionized water (with 0.1% of Triton X to reduce agglomeration) in a concentration of ∼ 0.001 vol%.

An HPLC pump (Waters, model 515) was used to pump the samples through the devices at a controlled flow rate with a read out of the pressure.

During the operation, the devices were observed with an inverted fluorescence microscope (Olympus IX73 with an Orca-Flash 4.0 LT digital CMOS camera). Images were taken with a magnification of 20X and a 2 s exposure time. The intensity profiles were analysed by ImageJ.

### Experimental evaluation of ${{\varvec{Q}}}_{{\varvec{m}}{\varvec{a}}{\varvec{x}}}$

The conditions that lead to ${F}_{D}$ surpassing ${B}_{L}$ were explored experimentally with a set of devices with the same cross section  (41 × 84 µm ($WxH$, measured values)), each consisting of one inlet, two loops with similar $R$ and one outlet. The only variable that changed between devices was $R$, which ranged from 40 mm to 2.5 mm in a geometrical proportion of $\sqrt[4]{2}$, making a total of 17 devices. The microchannels were dry etched on silicon to avoid a possible deformation of the systems with the flow.

We mapped ${Q}_{max}$ on a plot with $Q$ in the Y axis and $R$ in the X axis. For that, we ramped up $Q$ while observing the outlet and considered ${B}_{L}$ to be breached when the intensity near the outer wall started to increase and was ~ 2.5% of that near the inner wall. The analysis of the intensity was done with ImageJ, with a prior reduction of the noise by the command “Subtract background” with a rolling ball of 50 pixels (~ 16 µm). Particle sizes 8, 6, 4.8, 4, 3.2 and 2.2 µm in diameter were used.

The results with this first set of devices reflected the influence of ${U}_{m}$, $a$ and $R$. To include $W$, we fabricated another set of devices with a cross section four times smaller (10.5 × 22 µm ($WxH$, measured values)) and $R$ ranging from 5 mm to 0.6 mm in a geometrical proportion of $\sqrt[3]{2}$, making a total of 10 devices. ${Q}_{max}$ was mapped in a similar way for such systems, using particle sizes 2.2, 1.0, 0.92, 0.79 and 0.7 µm in diameter.

### Simulations

Simulations of the fluid flow were performed in microchannels with different cross sections using COMSOL Multiphysics v.5.5 (Laminar Flow interface; Navier–Stokes in a 3D space) in order to understand the secondary flow ($${U}_{D}$$). The flow was solved for water at room temperature in HARC microchannels extending a quarter of a loop. The flow rate was set at one end (inlet) as fully developed flow, and the pressure was set to zero at the other end (outlet). The secondary flow was analysed at a cross section ~ 2/3 of the channel length from the inlet to ensure a fully developed flow. The mesh generation was set to physics-controlled mesh and the maximum size of the elements was set to $$W$$/30. With the results, the strength of the secondary flow at the ROI in HARC systems ($${U}_{D,ROI})$$ was obtained and, with it, an analytical expression for the drag that the secondary flow causes on particles ($${F}_{D,ROI}$$) was derived by assuming a Stokes drag ($${F}_{D,ROI}=3\pi \mu a{U}_{D,ROI}$$).

### Expression for ${{\varvec{B}}}_{{\varvec{L}}}$

The conditions obtained experimentally for ${Q}_{max}$ represent the particular situation where ${F}_{D, ROI}$ surpasses ${B}_{L, ROI}$. Introducing them into the analytical expression for ${F}_{D, ROI}$ was used to derive an expression for ${B}_{L, ROI}$.

### Definition of the upper limit in HARC systems

The obtained expressions for ${F}_{D, ROI}$ and ${B}_{L, ROI}$ were substituted in Eq. (3), thereby defining analytically the upper limit where HARC systems focus particles.

## Results and discussion

### Characterization of ${{\varvec{F}}}_{{\varvec{D}},\boldsymbol{ }{\varvec{R}}{\varvec{O}}{\varvec{I}}}$ with COMSOL Multiphysics

**Figure 5:**
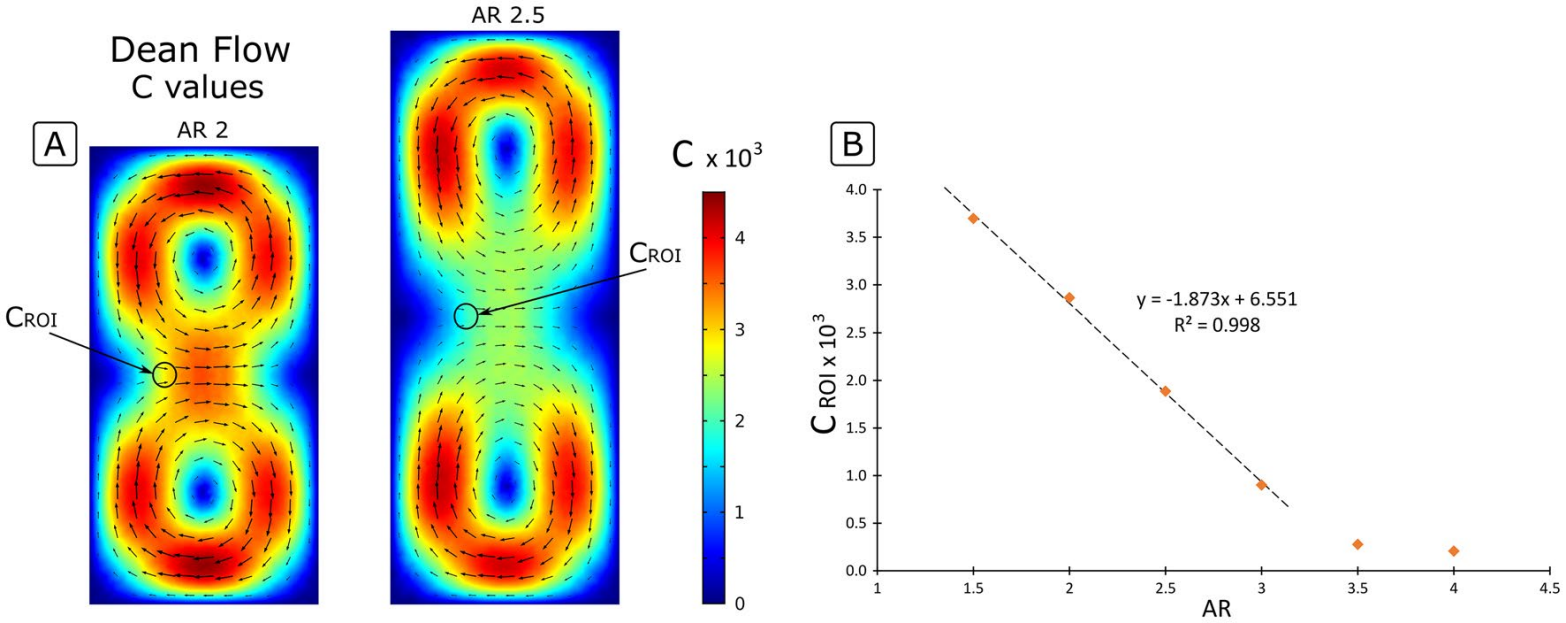
(**A**) Secondary flow (${U}_{D}$) in HARC systems with 2 and 2.5 normalized by $\frac{\rho }{\mu }{U}_{m}^{2}{W}^{2}/R$. The color represents the local strength of the vortexes ($C$ value). (**B**) *C* value at the *region of interest* (${C}_{ROI}$) for different $AR$.

COMSOL Multiphysics was used to simulate the flow in HARC microchannels with rectangular cross sections and different $AR$. Interestingly, a given cross section results in a secondary flow with a characteristic shape that is largely invariant, while the magnitude (${U}_{D}$) varies and scales at each point as ${U}_{D}\sim \frac{\rho }{\mu }{U}_{m}^{2}{W}^{2}/R$, as first reported by Dean^24,27^. One can then express ${U}_{D}=C\frac{\rho }{\mu }{U}_{m}^{2}{W}^{2}/R$, where $C$ is introduced as a coefficient that adjusts the magnitude of ${U}_{D}$ to the local position within the cross section and that depends on its geometry. With the results of the simulations (${U}_{D}$) for different rectangular cross sections, 2D maps of $C$ were obtained by plotting $C={U}_{D}/\left(\frac{\rho }{\mu }{U}_{m}^{2}{W}^{2}/R\right)$; two examples are shown in Fig. 5A. These maps may be regarded as a normalized secondary flow for a given cross section, whose shape remains invariant while its strength scales with $\rho , \mu , {U}_{m}, W$ and $R$, as explained before. The value of $C$ at the ROI (${C}_{ROI}$), which is the point of interest in this study, strongly depended on the $AR$, as shown in Fig. 5B. An equation was fitted for ${C}_{ROI}=f(AR)$, obtaining an expression for the velocity of the secondary flow at the ROI (${U}_{D, ROI}$) in HARC systems (Eq. 4):

4 $${U}_{D, ROI}\approx {C}_{ROI}\frac{\rho }{\mu }\frac{{U}_{m}^{2}{W}^{2}}{R}$$

with ${C}_{ROI}=\left(6.55-1.87(AR)\right) {10}^{-3}$ being accurate at least for $AR$ between 1.5 and 3, which is the practical range of interest.

The drag force exerted by the secondary flow was calculated as a Stokes drag ${F}_{D}=3\pi \mu a{U}_{D}^{*}$; where ${U}_{D}^{*}$ is the relative speed of the particle compared to speed of the secondary flow. In the scenario where particles are focused, the relative speed is maximum; ${U}_{D}^{*}={U}_{D,ROI}$, and ${F}_{D, ROI}$ becomes:

5 $${F}_{D, ROI}=3\pi a {C}_{ROI} \rho \frac{{U}_{m}^{2}{W}^{2}}{R}$$

With Eq. (5), the left side of Eq. (3) is defined, leaving the study of ${B}_{L}$ to complete the equation.

### Characterization of ${{\varvec{Q}}}_{{\varvec{m}}{\varvec{a}}{\varvec{x}}}$

**Figure 6:**
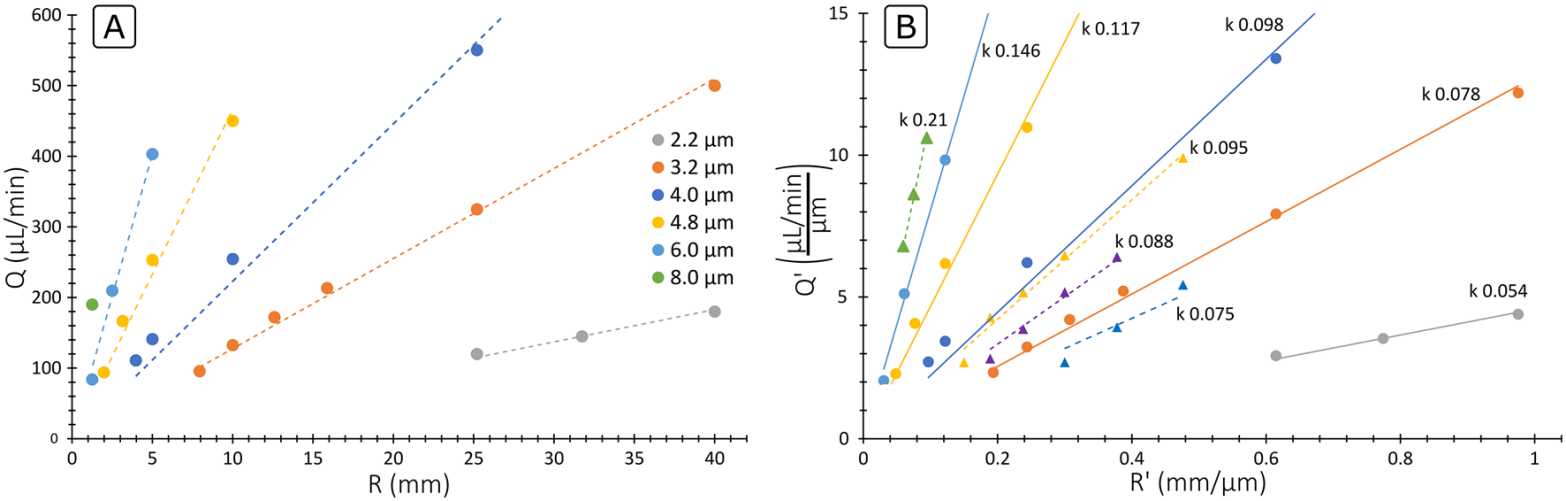
(**A**) Experimental data of ${Q}_{max}$ obtained with microchannels consisting of two loops, fixed $R$ and 41 × 84 µm ($WxH$). (**B**) Experimental data of ${Q}_{max}$ from both sets expressed with generalized variables, ${Q}^{\mathrm{^{\prime}}}=Q/W$, ${R}^{\mathrm{^{\prime}}}=R/W$ and $k=a/W$. The data marked with circles corresponds to the set with cross Section. 41 × 84 µm ($WxH$) and the data marked with triangles to the set with cross section 10.5 × 22 µm ($WxH$).

Figure 6A shows the experimental results obtained for ${Q}_{max}$ with the first set of devices (two loops, fixed $R$, 41 × 84 µm ($WxH$)). Each device allowed for the exploration of a vertical line on the graph; the flow rate was ramped up and the conditions where particles stopped being focused were marked. The transition was sharp and clear for $Q>{Q}_{min}$ (~ 100 µL/min for said cross section and number of loops), as particles transitioned from being focused with high quality to not focusing. For a given $Q$, smaller particles needed much larger $R$ (weaker ${F}_{D}$) to remain focused, stemming from the known fact that ${F}_{L}$ is weaker for smaller sizes. For the same reason, for a given $R$, larger particles remained focused up to higher $Q$.

The plot was generalized by dividing the variables by $W$; obtaining ${Q}^{^{\prime}}=Q/W$, ${R}^{^{\prime}}=R/W$ and $k=a/W$. Figure 6B shows the data from both sets of devices together (41 × 84 µm and 10.5 × 22 µm) in such plot. Despite the scaling factor of four between them, all data fitted well and showed the same trend; see how the different lines obtained for all $k$ values are well ordered and follow a linear trend.

**Figure 7:**
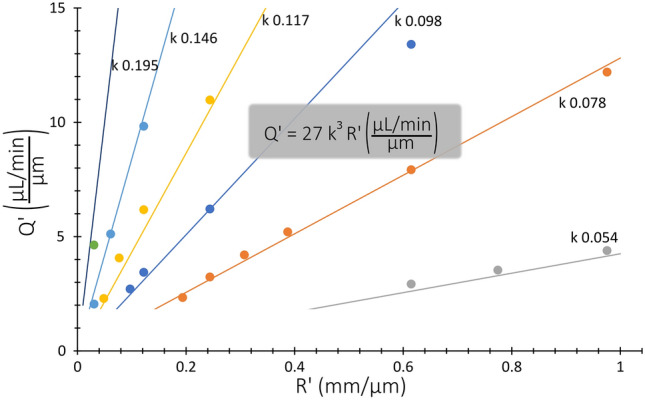
Analytical expression for ${Q}_{max}^{\mathrm{^{\prime}}}$ (Eq. 6, straight lines) together with experimental data (dots) from the first set (41 × 84 µm ($WxH$)) for different $k$ values ($k=a/W$).

The data from the first set was used to find the underlying pattern between ${Q}^{^{\prime}}$ and ${R}^{^{\prime}}$. In Fig. 7, lines following Eq. (6) are plotted together with the experimental data.

6 $${Q}_{max}^{^{\prime}}=27 {k}^{3}{R}^{^{\prime}}\frac{\mu L/min}{\mu m}$$

It can be seen that the agreement is remarkable given the simplicity of the equation. Note that, although both sets show the same trend, only the first one was used to find a fit because the relative errors in the small devices are expected to be larger, which is intrinsic to the technologies used for the fabrication; the possible lithography errors (hundreds of nm) and the roughness of the sidewalls by dry etching are the same in both cases, but the impact is much bigger for a small system. Equation (6) can therefore be used to predict the line of ${Q}_{max}$ with good accuracy for HARC systems with $AR$ 2.05.

Finally, as it will be explained in the next sections, Eq. (6) can be extended for any $AR$ with help of Eq. (4), obtaining:

7 $${Q}_{max}=L {k}^{3}R$$

where $$L=\frac{72AR}{{C}_{ROI}{K}_{U}} \frac{\mu L/min}{mm}$$ and $${K}_{U}=2.26-0.13(AR)$$.

With Eq. (7) defining the upper limit ${Q}_{max}$, together with Eq. (2) defining the lower limit ${Q}_{min}$, every piece to engineer HARC systems for particle focusing was obtained. Bringing them together, a working range of flow rate where a HARC system focuses particles is defined:

8 $$\frac{0.6{AR}^{3}W}{ {N}_{L}}<Q<L {k}^{3}R$$

Note that Eq. (6) was obtained from experimental data that covered the range $Re$ 30 to 240 (with $Re$ calculated using ${U}_{max}$). The approximation may be part of a more complex trend and not be valid for lower or higher $Re$ values.

### Study of the lift barrier and relation with the lift force

From a practical point of view, Eq. (6) expresses the upper limit of $Q$ over which particles breach the Lift Barrier of a microchannel with $AR$ 2.05. But further than that, the equation contains the information of the particular event where ${F}_{D, ROI}$ surpasses ${B}_{L, ROI}$. Given that the expression for ${F}_{D, ROI}$ is known (Eq. 5), it can be used to derive an expression for ${B}_{L, ROI}$.

Equation (6) can be re-arranged so that ${F}_{D, ROI, AR 2.05}$ (Eq. 5 for $AR$ 2.05) appears on the left side:

9 $${F}_{D,ROI, AR\, 2.05}<J \rho \frac{{{U}_{m}a}^{4}}{{W}^{3}}$$

where $J=3.6 \pi {10}^{-6}\frac{{m}^{2}}{s}$ is the Lift Barrier constant. See ESI Sect. 2 for a stepwise derivation.

By analogy with Eq.( 3) (${F}_{D, ROI}<{B}_{L, ROI}$), the right term of Eq. (9) is an analytical expression for ${B}_{L, ROI}$:

10 $${B}_{L, ROI}=J \rho \frac{{{U}_{m}a}^{4}}{{W}^{3}}$$

Note that Eq. (10) is independent of $AR$. This fact was expected since ${B}_{L}$ is born from the main flow and, at the symmetry line, this last is similar to a flow between two infinite parallel planes (Poiseuille flow, defined by ${U}_{m}$ and $W$). In fact, assuming the dominant transversal force is ${F}_{L}$, ${B}_{L, ROI}$ coincides with the ${F}_{L}$ induced on a particle in a Poiseuille flow at $W/3$ from the walls, being ${F}_{L}=f({U}_{m}, W, a)$, as described by Ho & Leal^4^ in 1974. Therefore, the expression for ${B}_{L, ROI}$ obtained experimentally in this paper can be considered as an expression for ${F}_{L}$ at the ROI:

11 $${F}_{L, ROI}=J \rho \frac{{{U}_{m}a}^{4}}{{W}^{3}}$$

with an experimentally measured scaling of ${F}_{L}$ for $Re$ between 30 and 240:

12 $${F}_{L}\sim \rho \frac{{{U}_{m}a}^{4}}{{W}^{3}}$$

The scaling of the lift force, here indirectly measured experimentally, points in a very interesting direction. The scaling was originally thought to be ${F}_{L}\sim \rho {{U}_{m}}^{2}{a}^{4}/{W}^{2}$, as proposed by Ho and Leal^4^ in 1974, obtained analytically for very low $Re$ numbers and $a\ll W$. Thereafter, the theory was extended for higher $Re$ numbers and particle sizes in the order of $W$. With it, it was observed analytically by Asmolov^5^ and via simulations by Liu^21^ that the proposed equation is essentially right but is to be corrected as $Re$ is increased. A lift coefficient (${c}_{L}$), which decreases as $Re$ increases, was then included, leaving ${F}_{L}={c}_{L}\rho {{U}_{m}}^{2}{a}^{4}/{W}^{2}$. Experimental evidence of this trend, where ${F}_{L}$ does not grow as strongly as ${F}_{L}\sim {{U}_{m}}^{2}$, has also been reported in multiple occasions^14,20,23^. However, still nowadays there is no consensus about the lift coefficient and different researchers point in different directions (Asmolov^5^, Di Carlo^7^, Zhou^23^, Liu^8,21^, Hood^9^). Our experimental results here presented also align with the trend mentioned above. The obtained expression for ${F}_{L}$ (Eq. 11) can be regarded as the inclusion a lift coefficient in Ho & Leal’s equation that is inversely proportional to the $Re$ number; ${c}_{L}\sim 1/Re\sim 1/({U}_{m}W)$. This is of great interest, as it simplifies the equation and eliminates the uncertainty arising from the controversy of the lift coefficient. In fact, this reminds of a well-known situation: the drag force, which scales as ${{c}_{D}U}^{2}$ (with ${c}_{D}$ being the drag coefficient), but at low $Re$, ${c}_{D}\sim 1/Re$ and the drag is known as Stokes drag, leaving ${F}_{D}\sim U$. Our results indicate a same behaviour of the lift force; ${F}_{L}\sim {{c}_{L}U}^{2}$ with ${c}_{L}\sim 1/Re$ at low $Re$.

Furthermore, ${F}_{L}\sim U$ agrees with the experimental evidence presented by Zhou^23^ in 2013, where the channel length needed for particles to reach the equilibrium perimeter in straight channels was observed to be invariant with the flow rate up to moderate $Re$ numbers ($Re$ 80 as reported by them, corresponding to $Re$ 160 here—they used the mean flow velocity for the definition of $Re$ while here we use the maximum flow velocity). An invariant length with $Q$ suggests that the particle migration velocity (${U}_{p}$) grows linearly with the main flow velocity ($U$). And given that ${U}_{p}\sim {F}_{L}/3\pi \mu a$, their finding also indicates ${F}_{L}\sim U$. However, the authors made a different interpretation of the events motivated by the fact that for higher $Re$ they observed an increase in the focus length. We believe that this increased length was not related to ${c}_{L}$ but rather to an expansion of the microchannels with the growing pressures needed as the flow rate is increased. With an expansion in $W$, certainly ${F}_{L}$ weakens and it takes longer for particles to migrate. This may originate in the fact that the channels used in said experiments were fabricated with PDMS. In the present work, by the use of silicon microchannels, this possible problem was eliminated and the linear trend was observed further—up to $Re\sim 240$.

### Generalization of the upper limit in HARC systems

Finally, introducing Eqs. (5) and (10) into Eq. (3), the condition to be fulfilled for particles not to cross the Lift Barrier is generalized for any $AR$:

13 $$ \begin{gathered} F_{D, ROI} < B_{L, ROI} \hfill \\ 3\pi a C_{ROI} \rho \frac{{U_{m}^{2} W^{2} }}{R} < J \rho \frac{{U_{m} a^{4} }}{{W^{3} }} \hfill \\ 1 < \frac{J}{{3\pi C_{ROI} }}\frac{{a^{3} R}}{{U_{m} W^{5} }} \hfill \\ \end{gathered} $$

Re-organizing Eq. (13) to have practical experimental variables:

14 $$ \begin{aligned} & Q < L k^{3} R \\ & Q_{max} = Lk^{3} R \\ \end{aligned} $$

where we coin $L=\frac{72(AR)}{{C}_{ROI}{K}_{U}}\frac{\mu L/min}{mm}$ as the HARC Limit coefficient. See ESI Sect. 3 for a stepwise derivation.

With Eq. (14), we obtain the final expression for the upper limit of flow rate in HARC systems (${Q}_{max}$) with $AR$ between 1.5 and 3.

To summarize, in this paper we identified a critical position of the cross section of HARC microchannels where the balance of the lift force and the drag by the secondary flow defines if the system focuses the particles or not (*region of interest,* ROI). Analytical expressions for the calculation of the forces (Eqs. 5 and 11) and their balance (Eq. 13) at said position are also provided. With this, the upper limit of flow rate of HARC systems is defined (Eq. 14) and, together with an expression for a lower limit (Eq. 1), it allows for an easy engineering of HARC systems for focusing particles. The experimentally measured strength of the lift force (Eq. 11) revealed a practical scaling law (Eq. 12), which we believe contributes to a better understanding of the phenomenon and may provide an interesting insight for the community.

### Limitations

From a practical point of view, Eqs. (13–14) describe the upper limit in HARC microchannels and allow for engineering the systems in a simple manner and with little uncertainty. On the other hand, while the observed linearity seems a good approximation for the range of $Re\sim 30-240$, the underlying physics may have a more complex behaviour outside this range—question that we leave for others to explore.

From a theoretical point of view, the experimental observations were related to the lift force and an expression revealing its strength and scaling was derived (Eq. 11). While the expression points in a reasonable direction and agrees with other reported experimental observations, it was mathematically derived by assuming a Stokes drag on the particles from the secondary flow, which is already a simplification. Also, the lift force accounted here is a net lift force; it comprises the summation of all the effects that induce a transversal force (except for the secondary flow) such as the Saffman and Magnus forces—see reviews for further information about these effects^1,2^.

The Lift Barrier constant was obtained with water; the only fluid used in these experiments. The dimensions $J\left[\frac{{m}^{2}}{s}\right]$ suggest that it may contain information about the density and the viscosity of the fluid—$J\sim \mu /\rho ?$ Further experiments including variations in these parameters may complement this study. If $J\sim \mu /\rho$, it would mean that, at low $Re$, ${F}_{L}$ depends on $\mu$ and not $\rho$, just as the drag force does.

Last, HARC systems provide the means to focus a range of particle sizes together in a stable line; ideal for laser interrogation and concentration. However, precisely because of such a feature, the systems lose one of the key capabilities of inertial focusing, the possibility to separate particles. We are, nevertheless, working on solving this and we will present the results in the near future.

## Conclusions

With this work, the description of HARC systems for focusing particles is completed. The systems must be engineered to operate between two limits. The lower one was previously defined and ensures that particles have time to reach the focus position. The upper one, which is developed here based on experimental evidence, sets the limit where the lift force is no longer strong enough to stop particles from following the secondary flow and the system fails to focus them.

Expressions for the design of HARC systems that allow for a high quality, single and stable position particle focusing are provided. Of special interest is the measured magnitude and scaling of the lift force, which may provide a valuable insight for the community.

We believe that HARC systems, with an intuitive focusing mechanism and two simple equations to achieve a stable focus, make the technology of inertial focusing and its excellent performance easily accessible, which may facilitate its implementation outside research laboratories.

## Supplementary Information

Supplementary Information

## Data Availability

Detailed mathematical derivations available in the Supplementary Information.

## References

[1] Martel JM, Toner M (2014). Inertial focusing in microfluidics. Annu. Rev. Biomed. Eng..

[2] Zhang J (2016). Fundamentals and applications of inertial microfluidics: A review. Lab Chip.

[3] Chung AJ (2019). A minireview on inertial microfluidics fundamentals: inertial particle focusing and secondary flow. Biochip J..

[4] Ho BP, Leal LG (1974). Inertial migration of rigid spheres in two-dimensional unidirectional flows. J. Fluid Mech..

[5] Asmolov ES (1999). The inertial lift on a spherical particle in a plane poiseuille flow at large channel Reynolds number. J. Fluid Mech..

[6] Matas JP, Morris JF, Guazzelli E (2004). Lateral forces on a sphere. Oil Gas Sci. Technol. IFP.

[7] Di Carlo D, Edd JF, Humphry KJ, Stone HA, Toner M (2009). Particle segregation and dynamics in confined flows. Phys. Rev. Lett..

[8] Liu C, Xue C, Sun J, Hu G (2016). A generalized formula for inertial lift on a sphere in microchannels. Lab Chip.

[9] Hood K, Lee S, Roper M (2015). Inertial migration of a rigid sphere in three-dimensional Poiseuille flow. J. Fluid Mech..

[10] Segré G, Silberberg A (1961). Radial particle displacements in poiseuille flow of suspensions. Nature.

[11] Warkiani ME (2016). Ultra-fast, label-free isolation of circulating tumor cells from blood using spiral microfluidics. Nat. Protoc..

[12] Zhou J (2019). Isolation of circulating tumor cells in non-small-cell-lung-cancer patients using a multi-flow microfluidic channel. Microsyst. Nanoeng..

[13] Cruz J (2017). High pressure inertial focusing for separating and concentrating bacteria at high throughput. J. Micromech. Microeng..

[14] Cruz J, Graells T, Walldén M, Hjort K (2019). Inertial focusing with sub-micron resolution for separation of bacteria. Lab Chip.

[15] Amini H, Lee W, Di Carlo D (2014). Inertial microfluidic physics. Lab Chip.

[16] Gou Y, Jia Y, Wang P, Sun C (2018). Progress of inertial microfluidics in principle and application. Sensors.

[17] Stoecklein D, Di Carlo D (2019). Nonlinear microfluidics. Anal. Chem..

[18] Liu, N., Petchakup, C., Tay, H. M., Li, K. H. H. & Hou, H. W. Spiral inertial microfluidics for cell separation and biomedical applications. In 99–150 (Springer, Singapore, 2019). 10.1007/978-981-13-6229-3_5

[19] Martel JM, Toner M (2013). Particle focusing in curved microfluidic channels. Sci. Rep..

[20] Cruz J, Hjort K, Hjort K (2021). Stable 3D inertial focusing by high aspect ratio curved microfluidics. J. Micromech. Microeng..

[21] Liu C, Hu G, Jiang X, Sun J (2015). Inertial focusing of spherical particles in rectangular microchannels over a wide range of Reynolds numbers. Lab Chip.

[22] Chun B, Ladd AJC (2006). Inertial migration of neutrally buoyant particles in a square duct: an investigation of multiple equilibrium positions. Phys. Fluids.

[23] Zhou J, Papautsky I (2013). Fundamentals of inertial focusing in microchannels. Lab Chip.

[24] Squires TM, Quake SR (2005). Microfluidics: fluid physics at the nanoliter scale. Rev. Mod. Phys..

[25] Schonberg JA, Hinch EJ (1989). Inertial migration of a sphere in Poiseuille flow. J. Fluid Mech..

[26] Matas JP, Morris JF, Guazzelli É (2004). Inertial migration of rigid spherical particles in Poiseuille flow. J. Fluid Mech..

[27] Dean WR (1927). XVI. Note on the motion of fluid in a curved pipe. Lond. Edinburgh Dublin Philos Mag. J. Sci..

