# Supplementary material for: The upper limit and lift force within inertial focusing in high aspect ratio curved microfluidics
Source: Sci Rep. 2021 Mar 19;11:6473. doi: 10.1038/s41598-021-85910-2 (PMC7979744; doi:10.1038/s41598-021-85910-2)
Supplement: Supplementary file 1 — Supplementary Information [file 41598_2021_85910_MOESM1_ESM.pdf]

Supplementary Information

Main text: Upper Limit and Lift Force within Inertial Focusing in High Aspect Ratio Curved Microfluidics

Authors:

Javier Cruz[a] javier.cruz.mst@gmail.com
Klas Hjort[a] klas.hjort@angstrom.uu.se

[a] Division of Microsystems Technology, Uppsala University, Ångström Laboratory, Uppsala, Sweden

1. Background information

$$Q < 27\left(\frac{\mu L/min}{\mu m}\right)k^3 R \tag{ESI 1}$$

Equation ESI 1, obtained experimentally for AR 2.05 in the main text, reflects the upper limit for HARC systems with AR 2.05; the conditions for particles to remain focused by the inner wall or, in other words, for the force exerted on a particle by the secondary flow (F_D) not to surpass the Lift Barrier (B_L). Expressed in terms of forces:

$$F_{D,ROI} < B_{L,ROI} \tag{ESI 2}$$

Therefore, understanding the magnitude of F_D leads to understanding B_L. In the following paragraphs we will re-arrange Eq. ESI 1 into Eq. ESI 2 for the particular case of AR 2.05 and obtain an expression for B_L.

We will use the subindex ROI to indicate that the analysis refers to the *Region of Interest* in the cross section and the subindex AR 2.05 to indicate that the variable corresponds to a microchannel with AR 2.05.

Equations to have present:

- Dean flow at the ROI for any AR:

$$U_{D,ROI} \approx C_{ROI}\frac{\rho}{\mu}\frac{U_m^2 W^2}{R} \tag{ESI 3}$$

  With $C_{ROI} = (6.55 - 1.87AR)\ 10^{-3}$.
- Dean force at the region of interest for any AR:

$$F_{D,ROI} = 3\pi\mu a U_{D,ROI} \tag{ESI 4}$$

- Flow rate Q as function of the mean velocity ($\overline{U}$), W and AR:

$$Q\ = \overline{U}\, W^2 AR \tag{ESI 5}$$

- Relation between $\overline{U}$ and U_m:

$$U_m = K_U \overline{U} \tag{ESI 6}$$

where $K_U = (2.26 - 0.13AR)$ is a coefficient obtained based on COMSOL simulations; see Fig. ESI 1. It is common to find $K_U = 2$ in the literature for 3D flows and $K_U = 1.5$ for 2D flows. Indeed, such numbers are valid for circular pipes and for flows between two parallel infinite planes, respectively. However, in rectangular channels, the coefficient is close to that of a circular pipe for $AR \approx 2$ and tends to 1.5 as the AR becomes infinite. With $K_U = f(AR)$, we introduce a refinement of the coefficient, which is valid at least for AR 1.5-3.

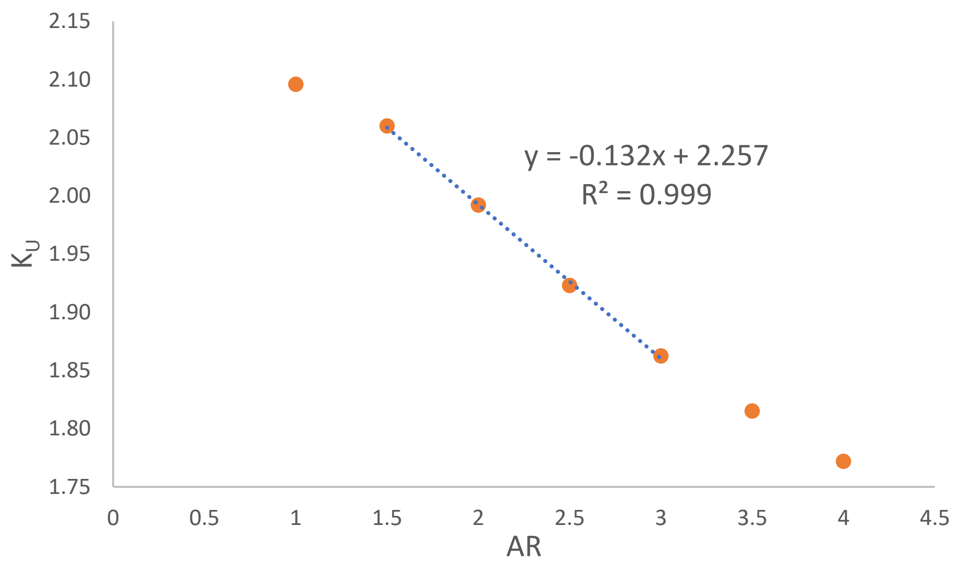

Figure ESI 1. Relation between the maximum flow velocity (U_m) and the mean flow velocity ($\overline{U}$) in microchannels with rectangular cross sections as function of the aspect ratio (AR). The relation $K_U = U_m/\overline{U}$ is generally approximated to $K_U \approx 2$ in 3D flows, which provides a good estimation. For more precise calculations, however, $K_U = (2.26 - 0.13AR)$ offers a refinement of the coefficient and is valid for AR between 1.5 and 3. The data was obtained from simulations by COMSOL Multiphysics.

2. Generalization of the experimental equation for the Upper Limit

Equation ESI 1 can be re-arranged to take the shape of Eq. ESI 3 and express $U_{D,ROI,AR\ 2.05}$ on the left side:

-In the place of Q, Eq. ESI 5 is introduced and Eq. ESI 6 substitutes $\overline{U}$ for U_m:

$$\frac{U_m}{K_{U,AR\ 2.05}} W^2 AR_{AR\ 2.05} < 27\ k^3 R\ \frac{\mu L/min}{\mu m}$$

$$\frac{U_m W^2}{R} < \frac{27}{60}\frac{K_{U,AR\ 2.05}}{AR_{AR\ 2.05}} k^3\ 10^{-3}\frac{m^2}{s} \qquad \text{(ESI 7)}$$

-Multiplying both sides by $C_{ROI,AR\ 2.05}\frac{\rho}{\mu}U_m$:

$$C_{ROI,AR\ 2.05}\frac{\rho}{\mu}\frac{U_m^2 W^2}{R} < C_{ROI,AR\ 2.05}\frac{\rho}{\mu}\frac{27}{60}\frac{K_{U,AR\ 2.05}}{AR_{AR\ 2.05}} U_m k^3\ 10^{-3}\frac{m^2}{s}$$

$$U_{D,ROI,AR\ 2.05} < C_{ROI,AR\ 2.05}\frac{\rho}{\mu}\frac{27}{60}\frac{K_{U,AR\ 2.05}}{AR_{AR\ 2.05}} U_m k^3\ 10^{-3}\frac{m^2}{s} \qquad \text{(ESI 8)}$$

-Bringing in the particular values for AR 2.05 ($C_{ROI,AR\ 2.05} = 2.78 * 10^{-3}$, $AR_{AR\ 2.05} = 2.05$, $K_{U,AR\ 2.05} = 1.99$), an expression for $U_{D,ROI,AR\ 2.05}$ is obtained, which reflects the velocity of the secondary flow that makes particles cross the Lift Barrier for HARC systems with AR 2.05.

$$U_{D,ROI,AR\ 2.05} < 1.2\ \frac{\rho}{\mu} U_m k^3 10^{-6} \frac{m^2}{s} \tag{ESI 9}$$

Knowing $U_{D,ROI,AR\ 2.05}$ and assuming a Strokes drag, the force induced by such flow can be calculated with Eq. ESI 4:

$$F_{D,ROI,AR\ 2.05} < 3.6\ \pi\ \rho\ \frac{U_m a^4}{W^3}\ 10^{-6} \frac{m^2}{s} \tag{ESI 10}$$

Eq. ESI 10 expresses the condition for F_D not to surpass B_L. By analogy with Eq. ESI 2, the right side of Eq. ESI 10 reflects the strength of $B_{L,ROI,AR\ 2.05}$:

$$B_{L,ROI,AR\ 2.05} = J\ \rho\ \frac{U_m a^4}{W^3} \tag{ESI 11}$$

where we coin $J = 3.6\ \pi\ 10^{-6} \frac{m^2}{s}$ as the Lift Barrier constant. Note that strength of the Lift Barrier at the ROI is not dependent on the AR. This fact was expected since B_L is born from the main flow and, at the symmetry line, this last is similar to that between two infinite parallel planes (defined by U_m and W). Therefore, although the strength of $B_{L,ROI}$ was obtained experimentally in microchannels with AR 2.05, Eq. ESI 11 is valid for any AR:

$$B_{L,ROI} = J\ \rho\ \frac{U_m a^4}{W^3} \tag{ESI 12}$$

Eq. ESI 12 is a milestone in understanding the HARC systems. Expressing the strength of the Lift Barrier, it allows the prediction of the conditions for HARC microchannels to focus particles.

Now that general expressions for both forces in play at the ROI are known, $F_{D,ROI}$ (Eq. ESI 4) and $B_{L,ROI}$ (Eq. ESI 12) can be substituted in Eq. ESI 2 to obtain the analytical expression for particles not to cross the Lift Barrier (Eq. ESI 13):

$$F_{D,ROI} < B_{L,ROI}$$

$$3\pi a C_{ROI}\ \rho \frac{U_m^2 W^2}{R} < J\ \rho\ \frac{U_m a^4}{W^3}$$

$$1 < \frac{J}{3\pi C_{ROI}} \frac{a^3 R}{U_m W^5} \tag{ESI 13}$$

3. Practical variables for microfluidic systems

Re-organizing Eq. ESI 13 to have practical experimental variables:

-Grouping terms:

$$U_m W^2 < \frac{J}{3\pi C_{ROI}} k^3 R \tag{ESI 14}$$

-And multiplying both sides by $\frac{AR}{K_U}$:

$$\frac{AR}{K_U}U_m W^2 < \frac{AR}{K_U}\frac{J}{3\pi C_{ROI}}k^3 R$$

$$Q < Lk^3 R \qquad \text{(ESI 15)}$$

where $L = J\frac{AR}{3\pi C_{ROI}K_U} = 1.2\frac{AR}{C_{ROI}K_U}10^{-6}\frac{m^2}{s}$ is the HARC Limit coefficient. In more practical units for microfluidics, $L = \frac{72AR}{C_{ROI}K_U}\frac{\mu L/min}{mm}$. Equation ESI 15 defines the final expression for the upper limit of flow rate in HARC systems (Q_{max}) with AR between 1.5 and 3.
